# Can early-onset acquired demyelinating syndrome (ADS) hide pediatric Behcet's disease? A case report

**DOI:** 10.3389/fped.2023.1175584

**Published:** 2023-06-23

**Authors:** Mattia Pozzato, Robertino Dilena, Greta Rogani, Gisella Beretta, Sofia Torreggiani, Stefano Lanni, Alessandra Tozzo, Francesca Andreetta, Paola Cavalcante, Fabio Triulzi, Filippo Martinelli Boneschi, Francesca Minoia, Giovanni Filocamo

**Affiliations:** ^1^ Neurology Unit & MS Centre, Fondazione IRCCS Ca’ Granda Ospedale Maggiore Policlinico, Milan, Italy; ^2^ Dino Ferrari Centre, Neuroscience Section, Department of Pathophysiology and Transplantation, University of Milan, Milan, Italy; ^3^ Neuropathophysiology Unit, Fondazione IRCCS Ca’ Granda Ospedale Maggiore Policlinico, Milan, Italy; ^4^ Pediatric Immunorheumatology Unit, Fondazione IRCCS Ca' Granda ospedale Maggiore Policlinico, Milan, Italy; ^5^ Infantile Neuropsychiatry Unit, Pediatric Neuroscience Department, IRCCS Fondazione Istituto Neurologico “C. Besta”, Milan, Italy; ^6^ Neurology 4 - Neuroimmunology and Neuromuscular Diseases Unit, Fondazione IRCCS Istituto Neurologico Carlo Besta, Milan, Italy; ^7^ Neuroradiology Unit, Fondazione IRCCS Ca’ Granda Ospedale Maggiore Policlinico, Milan, Italy; ^8^ Clinical Neurology, Department of Health Science CRC “Aldo Ravelli” for Experimental Brain Therapeutics, Hospital San Paolo ASST Santi Paolo e Carlo Milan and University of Milan, Milan, Italy

**Keywords:** Behcet’s disease, magnetic resonance imaging, children, early onset, acquired demyelinating syndromes (ADSs)

## Abstract

Behcet's disease (BD) is a rare vasculitis characterized by multisystemic inflammation. Central nervous system (CNS) involvement is rare and heterogeneous, particularly in the pediatric population. A diagnosis of neuro-Behcet could be highly challenging, especially if neurological manifestations precede other systemic features; however, its timely definition is crucial to prevent long-term sequelae. In this study, we describe the case of a girl who, at 13 months of age, presented with a first episode of encephalopathy compatible with acute disseminated encephalomyelitis, followed, after 6 months, by a neurological relapse characterized by ophthalmoparesis and gait ataxia, in association with new inflammatory lesions in the brain and spinal cord, suggesting a neuromyelitis optica spectrum disorder. The neurological manifestations were successfully treated with high-dose steroids and intravenous immunoglobulins. In the following months, the patient developed a multisystemic involvement suggestive of Behcet's disease, characterized by polyarthritis and uveitis, associated with HLA-B51 positivity. The challenge presented by this unique case required a multidisciplinary approach involving pediatric neurologists, neuro-radiologists, and pediatric rheumatologists, with all of these specialists creating awareness about early-onset acquired demyelinating syndromes (ADSs). Given the rarity of this presentation, we performed a review of the literature focusing on neurological manifestations in BD and differential diagnosis of patients with early-onset ADS.

## Introduction

Behcet's disease (BD) is an inflammatory vasculitis affecting the arteries or veins of all sizes and is characterized by cutaneous, ocular, articular, gastrointestinal, and/or central nervous system involvement. The first symptoms and signs of the disease commonly appear between the second and the fourth decade of life, but it can rarely develop in childhood and even in infancy (1).

Neurological involvement in pediatric BD is rare, but its early diagnosis is essential to prevent unfavorable outcomes. Central nervous system (CNS) manifestations can be attributed to two major pathways: first, and most commonly, through the development of an immune-mediated meningoencephalitis, which predominantly involves the brainstem, but can also involve the basal ganglia, thalamus, cortex, white matter, spinal cord, or cranial nerves; and second, as a consequence of thrombosis within the dural venous sinuses (2).

In this study, we describe the case of a very young girl who, at 13 months of age, presented the first neurological manifestation with radiologic features of acute disseminated encephalomyelitis (ADEM) and who subsequently developed a multisystemic involvement characterized by polyarthritis and uveitis suspicious for BD.

## Case report

**Figure 1:**
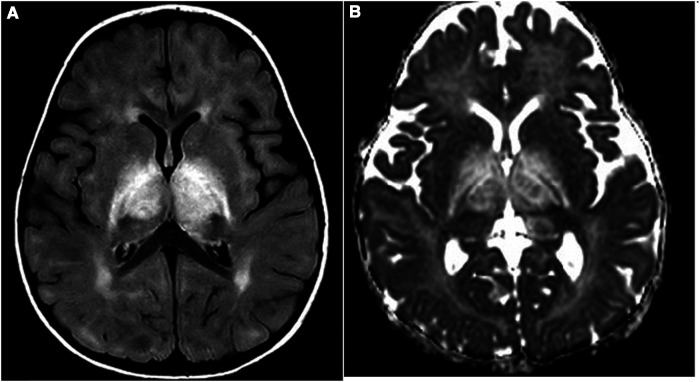
(**A,B**) First MRI study: FLAIR (**A**) and ADC (**B**) images show diffuse thalamic and internal capsule bilateral involvement with increased diffusion as for vasogenic edema.

A 13-month-old girl presented with an asymmetric weakness and hypertonia in the four limbs (both arms and legs), which appeared more severe on the right side than on the left side, with right head deviation. She could only perform some abnormal dystonic movements with the right limbs because her left limbs were almost plegic. She also presented with an altered state of consciousness, alternating periods of sleepiness with severe psychomotor agitation. Notably, 4 days before her neurological symptoms occurred, she presented with fever and cough, and 1 month prior to this, the patient had received vaccination against diphtheria, tetanus, pertussis, poliomyelitis, haemophilus B, hepatitis B, and pneumococcus. A cerebrospinal fluid (CSF) analysis revealed that the level was within the normal range, and a broad screening for infectious agents showed a negative result. The patient was also tested for the presence of oligoclonal bands, anti-AQP4, and anti-MOG antibodies. Brain magnetic resonance imaging (MRI) showed symmetrical thalamic lesions, with extension to the hemispheric white matter and to the right optic tract, without contrast enhancement (Figure 1A). A diagnosis of acute disseminated encephalomyelitis (ADEM) was made, and endovenous high-dose methylprednisolone therapy was started for 7 days, which involved a cycle of high-dose intravenous immunoglobulins (IVIg), leading to rapid clinical recovery and radiological response at the 3-month control brain MRI, without any radiological progression (Figure 1B). Due to the unusual coexistence of extrapyramidal dystonic manifestations at onset, thiamine-responsive basal ganglia disease (BTBGD) was excluded by sequencing the *SLC19A3* gene; likewise, inherited inborn errors of metabolism, including mitochondrial diseases, were excluded.

**Figure 2:**
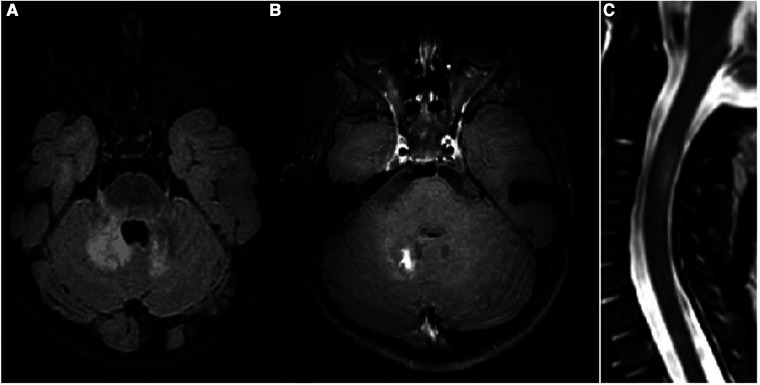
(**A–C**) Third MRI study: FLAIR (**A**) and postcontrast T1WI (**B**) of the cerebellum and a STIR midsagittal image of the cervical spine (**C**). New lesions on deep cerebellar white matter and V cranial nerves origin are visible, with a postcontrast enhancement of the right lesion. A new lesion in the cervical spine is also visible at the C3–C4 level.

Oral prednisone was tapered in 2 months. Four months later, the child reached the gait milestone, and a limping on the right leg was noticed, with an attitude in extra rotation of the right lower limb. One month later, and 17 days after the vaccination against measles, parotitis, and rubella, the patient presented with a second neurological episode characterized by the acute onset of gaze paralysis, left eye esophoria, vertical nystagmus, and inability to walk due to severe gait ataxia. The patient again underwent a brain and spinal cord MRI, which showed new lesions in the supra- and infratentorial white matter, optic nerves, some of which with gadolinium enhancement, and longitudinally extensive transverse myelitis (LTEM) lesion at the C2–C4 level, suggestive of neuromyelitis optica spectrum disorder (NMOSD) (Figure 2). Visual evoked potentials (EPs) showed an increase in P100 latency in both eyes, while brainstem auditory and somatosensory EP demonstrated an increased central conduction velocity. CSF analysis again showed that the level was within normal range, and no oligoclonal bands nor anti-AQP4 or anti-MOG antibodies were detected. However, a CSF cytokine panel assay showed multiple increases in proinflammatory cytokines, such as IL-6, IL-1Ra, MCP-1, and IP-10/CXCL10 (Supplementary Table S1). Blood tests showed antinuclear antibody (ANA) positivity (>1:640 titer) and the presence of HLA-B51. The patient was re-treated with high-dose steroids and monthly high-dose IVIg, which resulted in good neurological recovery, despite the persistence of a fluctuating limp in the right leg with a limited range of motion.

An MRI performed at 29 months of age did not show any new brain or spinal cord lesion but highlighted the presence of active arthritis of the right temporomandibular joint (Supplementary Figure S1). A complete joint assessment performed at that time by pediatric rheumatologists revealed swelling, pain, and limitation in the motion of the right knee and ankle. The presence of active arthritis was confirmed by an ultrasound assessment.

The very early onset of neurological manifestations and the subsequent development of polyarthritis led us to consider the wide spectrum of inherited diseases characterized by neuroinflammation. Type I interferon signature was negative. Whole-exome sequencing was then performed, with a particular focus on the genes involved in neuroinflammatory pathways (3) without any evidence of pathogenic variants.

Given the broad differential diagnosis performed, the presence of CNS parenchymal lesions located in the brain and spinal cord with a relapsing-remitting course, the good clinical recovery with immunomodulating treatment, and the evidence of polyarthritis associated with HLA-B51 positivity suggested a possible diagnosis of Bechet's disease with neurological involvement.

Intra-articular steroid injections were administered on the right knee and ankle, and therapy with colchicine (0.5 mg/daily) and methotrexate (7.5 mg/weekly) was started, with a stabilization of the neurological picture both clinically and radiologically. Due to the occurrence of tibiotarsal arthritis relapses, two more intraarticular steroid injections with the addition of biologic therapy with anti-IL6 (tocilizumab) were required for the girl at the age of 3 years and 3 months, which resulted in a very good clinical response and complete articular remission.

However, 3 years and 6 months from the disease onset, the patient developed bilateral uveitis despite ongoing treatment with tocilizumab and was, therefore, switched to adalimumab as part of anti-TNFα treatment, following which she showed a positive clinical and ophthalmological response. At the last follow-up, performed at the age of 5 years, 6 months after the start of adalimumab, the patient appeared fine with the disease and uveitis in remission.

## Discussion

Early-onset neurological manifestations are often a diagnostic challenge, especially when they are the first and isolated clinical features. In children, systemic symptoms may develop over the years and differential diagnosis covers a broad range of conditions, including systemic autoinflammatory/autoimmune disorders, acquired demyelinating syndromes (ADSs), and inherited autoinflammatory, neurological, and metabolic disorders. The case herein presented highlights this diagnostic dilemma.

ADSs represent a group of clinical conditions characterized by an inflammation of the CNS and consequent damage to the myelin. ADSs can be classified as follows: (1) monophasic forms, such as ADEM or clinically isolated syndrome (CIS); (2) polyphasic or chronic forms, including relapsing ADEM, multiple sclerosis (MS), NMOSD, or CNS involvement in the context of systemic inflammatory diseases, such as neuro-lupus, neuro-sarcoidosis, neuro-Bechet's, and others (4). A distinction needs to be made between monophasic demyelinating syndrome and polyphasic demyelinating syndrome, which is relevant for clinical management. In monophasic forms, steroids and IVIg are the treatments of choice, while chronic disorders often require disease-modifying therapy in order to prevent the accumulation of disability.

The complex workup in a pediatric patient with a suspicion of ADS is presented in Supplementary Figure S2), and the most common differential diagnoses are summarized and detailed in Table 1.

**Table 1:** Differential diagnosis in the context of acquired demyelinating syndromes in pediatric age with principal clinical characteristics.

	Pediatric ADEM (5–10)	Pediatric MS (11–19)	Pediatric NMO (20–28)	Pediatric Neuro-Behçet (29–34)
Clinical features	• Onset within days or weeks after a viral infection or immunization • Median age of onset 5–8 years • Typical presentation: altered mental status, multifocal neurological symptoms • Atypical presentation: stroke-like episodes, seizures, headache, emesis • 70%–90% monophasic vs. 10%–30% multiphasic • 65%–85% good functional recovery; 8% develop MS over time	• 3%–5% of all MS patients <16 years of age, <1% <10 years of age • Median age of onset 13 years • 98% RRMS (vs. 84% in adults) • More aggressive disease onset with disabling clinical symptoms and higher relapse rates • Slower disease progression (>10 years than adults) but moderate-to-severe disability reached at a younger age • Significant cognitive impairment	• 4% of all NMOSD • Median age of onset 10 years • Typical symptoms: visual and motor impairment and constitutional symptoms (vomiting, fever, intractable hiccups) • 90% relapsing-remitting • Poor outcome and higher EDSS	• 3%–30% of children with Behçet's disease • Median age of onset 11 years • Systemic manifestation: recurrent fever, oral and genital aphthosis, uveitis, venous and arterial thrombosis, skin lesions, gastrointestinal symptoms. • Neurological symptoms: chronic headaches, seizure, cranial nerve involvement, focal neurological signs, cognitive impairment • 75% neurological sequelae
MRI findings	• Multiple, bilateral, asymmetrical lesions • Poorly marginated • Involvement of the gray matter, in particular, the thalami or basal ganglia • Multiple and synchronous enhancement • Resolution in 3 months	• Periventricular lesions, perpendicular to the long axis of the corpus callosum • Ovoid lesions • Well-defined lesions • Black holes • Single and asynchronous enhancement	• Typical presentation: optic neuritis and/or longitudinally extensive transverse myelitis. • Other typical localizations: brainstem and fourth ventricle, diencephalon around the third ventricle and aqueduct, and periependymal lesions around the lateral ventricles.	• CNS non-parenchymal manifestations: cerebral venous thrombosis, cranial nerve palsy, meningeal involvement • CNS parenchymal manifestations: lesions that can affect the brainstem, basal ganglia, thalami, optic nerve, and bi-hemispheric white matter
Laboratory findings	• CSF analysis: oligoclonal bands rare • Anti-MOG antibodies with a high titer in the acute phase and a trend to decrease and normalize over time	• CSF analysis: oligoclonal bands in up to 92% of patients. • No specific antibodies	• CSF analysis: oligoclonal bands in 31% • Anti-AQP4 (70%) and anti-MOG (10%–15%) antibodies, 15%–20% double-seronegative	• Moderate elevation in acute phase reactants (ESR, CRP) • HLA-B51+ (40%–65% of patients with BD and in 10%–20% of healthy controls)
Therapies	• Steroids • IVIG • Plasma exchange	*First-line therapies* • Interferon beta • Glatiramer acetate • Dimethyl fumarate *Second-line therapies* • Natalizumab • Fingolimod • Rituximab • Cyclophosphamide	• Steroids • IVIG • Plasma exchange • Rituximab • Mycophenolate mofetil • Azathioprine	• Steroids • Colchicine • Azathioprine • Mycophenolate mofetil • TNFα inhibitors • Anti-IL1 (Anakinra) • Anti-IL6 (Tocilizumab)

ADEM, acute disseminated encephalomyelitis; MS, multiple sclerosis; NMO, neuromyelitis optica; RRMS, relapsing-remitting multiple sclerosis; NMOSD, neuromyelitis optica spectrum disorder; EDSS, expanded disability status scale; CNS, central nervous system; CSF, cerebrospinal fluid; MOG, myelin oligodendrocyte glycoprotein; AQP, aquaporin; ESR, erythrocyte sedimentation rate; CRP, C-reactive protein; ANA, antinuclear antibodies; ENA, extractable nuclear antigen; RF, rheumatoid factor; HLA, human leukocyte antigen; BD, Behcet disease; IVIG, intravenous immunoglobulins; TNF, tumor necrosis factor; IL, interleukin.

Our patient had a very early-onset neurological manifestation, exceptionally described so far in BD (35). Aphthosis was never reported in our patient; however, oral aphthosis is rarely present during the first years of age, even in patients with a full-blown diagnosis of BD in older age (29, 30).

As no single pathognomonic test exists, BD still represents a clinical challenge, particularly in the pediatric population, and its diagnosis relies on clinical findings and the exclusion of mimicking conditions. In our patient, neuro systemic lupus erythematosus (SLE) was considered. However, the absence of any cutaneous, hematological, or renal signs of the disease, the absence of specific antibodies (double-strained DNA), extractable nuclear antigen (ENA), or antiphospholipids, and the absence of complement consumption did not support this hypothesis. Furthermore, the extremely young age of the patient would have been unusual for classic SLE. Despite the intriguing monogenic SLE/interpheronopathy, the interferon gene signature was negative and the whole-exome sequencing performed excluded all genes related to those conditions. The normal level of the angiotensin-converting enzyme (ACE) and the absence of granulomatous lesions, particularly of granulomatous uveitis, made neuro-sarcoidosis unlikely.

Adult criteria for BD showed poor sensitivity and/or specificity in children (31, 32). In 2015, a consensus classification criteria set, based on a cohort of 219 children with BD, was established for pediatric BD (PEDBD) (29). The diagnosis of BD can be considered in the presence of three of the six following recurrent symptoms: oral aphthous lesions, genital ulcers, skin involvement, eye involvement, neurological findings, and vascular findings. However, clinical symptoms might develop over the years in patients with Behcet’s disease, hampering the application of the classification criteria in daily clinical practice.

The term neuro-Behcet disease (NBD) is used to define the nervous system involvement in BD. The rate of pediatric NBD was variably reported in the literature from 3% to more than 30% of children (30, 33). Neurological manifestations can precede other systemic features for a long time, making the diagnostic workup challenging. Early-onset BD is often insidious, due to its proteiform presentation, and its prompt diagnosis, which is essential to prevent adverse outcomes, is difficult, particularly when a rare manifestation, such as the neurological one, is the leading or the only clinical feature.

NBD can be classified into two major forms: (1) “non-parenchymal or vascular” disease, characterized by intracranial hypertension, typically due to dural sinus thrombosis and (2) “parenchymal” disease, which may include recurrent encephalomyelitis, aseptic meningitis, cranial nerve palsy, and neuropathy. Although rare, cerebral venous thrombosis is the most common presentation of pediatric NBD (1). MRI findings, although aspecific, are essential to guide differential diagnosis (34). CSF usually reveals elevated protein levels and pleocytosis. Recent studies have suggested the involvement of proinflammatory pathways such as IL-1, IL-6, IL-33, and INFγ in the CSF of patients with NBD (36). HLA-B51 positivity is considered the most important genetic risk factor of the disease and is described in 40%–65% of patients with BD but also in 10%–20% of healthy subjects; therefore, it should not be considered a diagnostic criterium; however, its positivity during the occurrence of compatible symptoms may support the diagnosis of BD.

In a case series of 26 children with BD, 11 developed neurological abnormalities with neurologic involvement at a mean age of 13.5 years, although not all fulfilled the criteria of BD at the onset. Two patients presented with a neurologic attack prior to the systemic manifestations of BD. One had parenchymal CNS involvement as well as oral aphthae at the age of 8 and fulfilled the International Study Group for Behcet Disease (ISGBD) criteria only many years later (37). In another recent case series, five pediatric patients diagnosed with NBD were described. The mean age reported was 15 years and oral ulcers were present in all patients (38). Table 2 summarizes the prevalence and the neurological manifestations of pediatric patients with BD and neurological involvement reported in the literature.

**Table 2:** Summary of the main features and prevalence of neurological involvement in the principal international cohorts of pediatric Behcet disease.

Main features of neurological manifestations in pediatric neuro-Behcet disease cohorts			
	Number of cases with neurological involvement	Median age at onset, expressed in years (min–max)	Neurological manifestations
Yavuz et al. (39)	2	12 (9–15)	Midbrain stroke syndrome characterized by ipsilateral fascicular third cranial nerve palsy with contralateral hemiataxia (Claude syndrome)
Tütüncü Toker et al. (38)	5	16 (12–17)	Seizure, myopathy, transverse myelitis, polyneuropathy, venous thrombosis, and facial nerve paralysis
Uluduz (37)	26	13.5 (8–16)	Headaches (24), Epileptic seizure (3), Ataxia (2), Pyramidal findings (3), Dysarthria (1)
Metreau-Vastel et al. (40)	12	11	venous thromboses (5), recurrent meningoencephalitis (2), rhombencephalitis (2), transverse myelitis (1), peripheral neuropathy (1) or psychiatric disturbances (1)
Moraleda-Cibrián et al. (35)	1	0,5	ophthalmoplegia, followed by ataxia and dysmetria.
Koné-Paut et al. (41)	2	12 (9–15)	psychiatric disturbances, amenorrhea, massive loss of weight, frontal headaches, vomiting, and blurred vision. Headaches, nausea, and memory loss
Prevalence of neurological involvement in the principal international cohorts of pediatric Behcet disease			
	Number of cases with neurological involvement/number of pediatric cases	Median age at onset, expressed in years (min–max)	Neurological manifestations
Ekici Tekin et al. (42)	11/72	10 (N/A)	7/11 patients had extraparenchymal disease and 4/11 patients had parenchymal disease. 6/11 patients had pseudotumor cerebri, 6/11 patients had papilledema, 4/11 patients had reversible vision loss, 2/11 patients had diplopia
Butbul Aviel et al.. 2020 (43)	30/205	11 (1–15,9)	N/A
Shahram et al. (44)	9/205	10.5 (1–16)	Cerebral venous thrombosis (1), recurrent meningoencephalitis (3), parenchymal involvement (5, two of them with convulsions).
Gallizzi et al. (30)	34/110	8.3* (N/A)	Headache (27), cranial nerve palsy (5), cranial neuropathy (2), aseptic meningitis (2), optic neuritis (2), peripheral neuropathy (1). Venous thrombosis occurred in two patients.
Nanthapisal et al. (45)	13/46	4.9 (0.04–15.71)	One patient developed seizures and 11 patients reported frequent (more than once a week) headaches. One patient gradually developed neurogenic bladder dysfunction and required suprapubic catheter insertion.
Atmaca et al. (46)	4/110	11,6 (1–16)	N/A
Kone-Paut et al. (47)	10/65	8,4* (0–16)	Meningitis (6/10), benign intracranial hypertension (3/10), hemiparesis or paraparesis (3) with spastic quadriparesis in one case, seizures (2), and peripheral neuropathy including sixth nerve palsy (2). Brain computed tomography scan and magnetic resonance imaging findings included ventricular dilatation and hyperintensity signals in the pons, brainstem, putamen, and upper medulla regions. Dural sinus thrombosis was observed in three patients.

*Mean; N/A,not available.

## Conclusion

Diagnosis and treatment of pediatric NBD are challenging because of its rarity and heterogeneous presentation, particularly when neurological involvement occurs early in infancy and precedes systemic signs and symptoms. A multidisciplinary approach, involving neurologists, neuroradiologists, and rheumatologists, is crucial to define the most probable diagnosis in a timely manner and start an appropriate treatment regimen as soon as possible.

Early-onset NBD could severely affect psychomotor development, and therefore, a timely diagnosis is important to prevent the possible accumulation of disability and poor outcomes in adulthood.

## Data Availability

The raw data supporting the conclusions of this article will be made available by the authors without undue reservation.

## Supplementary material

SUPPLEMENTARY FIGURE S1 A–B

Fourth MRI study on TMJ; STIR sequence (a) and post contrast T1WI acquisition (B) show an inflammation of the right TMJ with slight bone edema on STIR (a, arrow) and periarticular enhancement after contrast (b, arrow).

SUPPLEMENTARY FIGURE S2

Flow-chart of the diagnostic approach to pediatric patients with a first neurological episode suggestive for demyelinating disease.

## References

[1] Mora P Menozzi C Orsoni JG Rubino P Ruffini L Carta A. Neuro-Behçet’s disease in childhood: a focus on the neuro-ophthalmological features. Orphanet J Rare Dis. (2013) 8:18. 10.1186/1750-1172-8-18 23360593 PMC3567996

[2] Al-Araji A Kidd DP. Neuro-Behçet’s disease: epidemiology, clinical characteristics, and management. Lancet Neurol. (2009) 8(2):192–204. 10.1016/S1474-4422(09)70015-8 19161910

[3] McCreary D Omoyinmi E Hong Y Mulhern C Papadopoulou C Casimir M Development and validation of a targeted next-generation sequencing gene panel for children with neuroinflammation. JAMA Netw Open. (2019) 2(10):e1914274. 10.1001/jamanetworkopen.2019.14274 31664448 PMC6824223

[4] Carbonell CF Chitnis T. Inflammatory demyelinating diseases in children: an update. Minerva Pediatr. (2013) 65(3):307–23. 23685382

[5] Alves-Leon SV Veluttini-Pimentel ML Gouveia ME Malfetano FR Gaspareto EL Alvarenga MP Acute disseminated encephalomyelitis: clinical features, HLA DRB1*1501, HLA DRB1*1503, HLA DQA1*0102, HLA DQB1*0602, and HLA DPA1*0301 allelic association study. Arq Neuropsiquiatr. (2009) 67(3A):643–51. 10.1590/S0004-282X2009000400013 19722042

[6] Pohl D Alper G Van Haren K Kornberg AJ Lucchinetti CF Tenembaum S Acute disseminated encephalomyelitis: updates on an inflammatory CNS syndrome. Neurology. (2016) 87(9 Supplement 2):S38–S45. 10.1212/WNL.0000000000002825 27572859

[7] Berzero G Cortese A Ravaglia S Marchioni E. Diagnosis and therapy of acute disseminated encephalomyelitis and its variants. Expert Rev Neurother. (2016) 16(1):83–101. 10.1586/14737175.2015.1126510 26620160

[8] Tenembaum SN. Disseminated encephalomyelitis in children. Clin Neurol Neurosurg. (2008) 110(9):928–38. 10.1016/j.clineuro.2007.12.018 18272282 PMC7116932

[9] Callen DJA Shroff MM Branson HM Li DK Lotze T Stephens D Role of MRI in the differentiation of ADEM from MS in children. Neurology. (2009) 72(11):968–73. 10.1212/01.wnl.0000338630.20412.45 19038851

[10] Di Pauli F Mader S Rostasy K Schanda K Bajer-Kornek B Ehling R Temporal dynamics of anti-MOG antibodies in CNS demyelinating diseases. Clin Immunol. (2011) 138(3):247–54. 10.1016/j.clim.2010.11.013 21169067

[11] Alroughani R Boyko A. Pediatric multiple sclerosis: a review. BMC Neurol. (2018) 18(1):27. 10.1186/s12883-018-1026-3 29523094 PMC5845207

[12] Boiko A Vorobeychik G Paty D Devonshire V Sadovnick D, the UBC MS Clinic Neurologists. Early onset multiple sclerosis: a longitudinal study. Neurology. (2002) 59(7):1006–10. 10.1212/WNL.59.7.1006 12370453

[13] Renoux C Vukusic S Mikaeloff Y Edan G Clanet M Dubois B Natural history of multiple sclerosis with childhood onset. N Engl J Med. (2007) 356(25):2603–13. 10.1056/NEJMoa067597 17582070

[14] Ghezzi A Banwell B Boyko A Amato MP Anlar B Blinkenberg M Meeting review: the management of multiple sclerosis in children: a European view. Mult Scler. (2010) 16(10):1258–67. 10.1177/1352458510375568 20685764

[15] Tenembaum SN Banwell B Pohl D Krupp LB Boyko A Meinel M Subcutaneous interferon Beta-1a in pediatric multiple sclerosis: a retrospective study. J Child Neurol. (2013) 28(7):849–56. 10.1177/0883073813488828 23666046

[16] Makhani N Schreiner T. Oral dimethyl fumarate in children with multiple sclerosis: a dual-center study. Pediatr Neurol. (2016) 57:101–4. 10.1016/j.pediatrneurol.2016.01.010 26996405

[17] Ghezzi A Pozzilli C Grimaldi LME Brescia Morra V Bortolon F Capra R Safety and efficacy of natalizumab in children with multiple sclerosis. Neurology. (2010) 75(10):912–7. 10.1212/WNL.0b013e3181f11daf 20820002

[18] Chitnis T Arnold DL Banwell B Brück W Ghezzi A Giovannoni G Trial of fingolimod versus interferon Beta-1a in pediatric multiple sclerosis. N Engl J Med. (2018) 379(11):1017–27. 10.1056/NEJMoa1800149 30207920

[19] Vartzelis G Maritsi D Nikolaidou M Garouf A Kilidireas C. Rituximab as rescue therapy for aggressive pediatric multiple sclerosis. Case Rep Pediatr. (2019) 2019:1–3. 10.1155/2019/8731613 PMC6679848 31428499

[20] McKeon A Lennon VA Lotze T Tenenbaum S Ness JM Rensel M CNS aquaporin-4 autoimmunity in children. Neurology. (2008) 71(2):93–100. 10.1212/01.wnl.0000314832.24682.c6 18509092

[21] Chitnis T Ness J Krupp L Waubant E Hunt T Olsen CS Clinical features of neuromyelitis optica in children: US network of pediatric MS centers report. Neurology. (2016) 86(3):245–52. 10.1212/WNL.0000000000002283 26683648 PMC4733158

[22] Popescu BFG Lennon VA Parisi JE Howe CL Weigand SD Cabrera-Gómez JA Neuromyelitis optica unique area postrema lesions: nausea, vomiting, and pathogenic implications. Neurology. (2011) 76(14):1229–37. 10.1212/WNL.0b013e318214332c 21368286 PMC3068006

[23] Cacciaguerra L Meani A Mesaros S Radaelli M Palace J Dujmovic-Basuroski I Brain and cord imaging features in neuromyelitis optica spectrum disorders: MRI and NMOSD diagnosis. Ann Neurol. (2019) 85(3):371–84. 10.1002/ana.25411 30635936

[24] Jarius S Pellkofer H Siebert N Korporal-Kuhnke M Hümmert MV Ringelstein M. Cerebrospinal fluid findings in patients with myelin oligodendrocyte glycoprotein (MOG) antibodies. Part 1: results from 163 lumbar punctures in 100 adult patients. J Neuroinflammation. (2020) 17(1):261. 10.1186/s12974-020-01824-2 32883348 PMC7470615

[25] Waters P Jarius S Littleton E Leite MI Jacob S Gray B Aquaporin-4 antibodies in neuromyelitis optica and longitudinally extensive transverse myelitis. Arch Neurol. (2008) 65(7):913–9. 10.1001/archneur.65.7.913 18625857

[26] de Seze J. MOG-antibody neuromyelitis optica spectrum disorder: is it a separate disease? Brain. (2017) 140(12):3072–5. 10.1093/brain/awx292 29194504

[27] Wingerchuk DM Banwell B Bennett JL Cabre P Carroll W Chitnis T International consensus diagnostic criteria for neuromyelitis optica spectrum disorders. Neurology. (2015) 85(2):177–89. 10.1212/WNL.0000000000001729 26092914 PMC4515040

[28] Tenembaum S Yeh EA, Guthy-Jackson Foundation International Clinical Consortium (GJCF-ICC). Pediatric NMOSD: a review and position statement on approach to work-up and diagnosis. Front Pediatr. (2020) 8:339. 10.3389/fped.2020.00339 32671002 PMC7330096

[29] Koné-Paut I Shahram F Darce-Bello M Cantarini L Cimaz R Gattorno M Consensus classification criteria for paediatric Behçet’s disease from a prospective observational cohort: PEDBD. Ann Rheum Dis. (2016) 75(6):958–64. 10.1136/annrheumdis-2015-208491 26698843

[30] Gallizzi R Pidone C Cantarini L Finetti M Cattalini M Filocamo G A national cohort study on pediatric Behçet’s disease: cross-sectional data from an Italian registry. Pediatr Rheumatol Online J. (2017) 15(1):84. 10.1186/s12969-017-0213-x 29268757 PMC5740899

[31] Hu YC Chiang BL Yang YH. Clinical manifestations and management of pediatric Behçet’s disease. Clin Rev Allergy Immunol. (2021) 61(2):171–80. 10.1007/s12016-020-08809-2 32767171

[32] Batu ED Sonmez HE Sozeri B Butbul Aviel Y Bilginer Y Ozen S. The performance of different classification criteria in paediatric Behcet’s disease. Clin Exp Rheumatol. (2017) 35(Suppl 108):119–23. 28406761

[33] Koné-Paut I. Behçet’s disease in children: an overview. Pediatr Rheumatol Online J. (2016) 14:10. 10.1186/s12969-016-0070-z 26887984 PMC4758175

[34] Saltik S Saip S Kocer N Siva A Yalçinkaya C. MRI findings in pediatric neuro-Behçet’s disease. Neuropediatrics. (2004) 35(3):190–3. 10.1055/s-2004-815826 15248102

[35] Moraleda-Cibrián M Aracil-Martínez MA Ercilla-González G Ros-Viladoms J Cusí-Sánchez V González-Pascual E Neurobehçet de presentación precoz [Early onset Neurobehçet’s disease]. Rev Neurol. (2006) 42(3):150–2. 10.33588/rn.4203.2005139 16475136

[36] Hamzaoui K Borhani-Haghighi A Kaabachi W Hamzaoui A. Increased interleukin 33 in patients with neuro-Behcet’s disease: correlation with MCP-1 and IP-10 chemokines. Cell Mol Immunol. (2014) 11(6):613–6. 10.1038/cmi.2014.31 24909741 PMC4220833

[37] Uluduz D Kürtüncü M Yapıcı Z Seyahi E Kasapçopur Ö Özdoğan H Clinical characteristics of pediatric-onset neuro-Behçet disease. Neurology. (2011) 77(21):1900–5. 10.1212/WNL.0b013e318238edeb 22076549

[38] Toker RT Bodur M Bican Demir A Okan MS. Neuro-Behçet is a rare disease but should be considered in all kinds of neurological findings, even in childhood. Clin Exp Rheumatol. (2022) 40(8):1588–92. 10.1016/B978-0-7020-4088-7.00110-3 35894070

[39] Yavuz P Solmaz I Kaya UA Akgoz A Oguz KK Aytac S Claude syndrome in childhood associated with probable neuro-Behcet disease. Neuropediatrics. (2023) 54(1):82–7. 10.1055/s-0042-1759794 36564024

[40] Metreau-Vastel J Mikaeloff Y Tardieu M Koné-Paut I Tran TA. Neurological involvement in paediatric Behçet’s disease. Neuropediatrics. (2010) 41(5):228–34. 10.1055/s-0030-1269909 21210339

[41] Koné-Paut I Chabrol B Riss JM Mancini J Raybaud C Garnier JM. Neurologic onset of Behçet’s disease: a diagnostic enigma in childhood. J Child Neurol. (1997) 12:237–41. 10.1177/088307389701200402 9203064

[42] Ekici Tekin Z Çelikel E Aydin F Kurt T Sezer M Tekgöz N Juvenile Behçet’s disease: a tertiary center experience. Clin Rheumatol. (2022) 41(1):187–94. 10.1007/s10067-021-05896-0 34476647

[43] Aviel YB Batu ED Sözeri B Aktay Ayaz N Baba L Amarilyo G Characteristics of pediatric Behçet’s disease in Turkey and Israel: a cross-sectional cohort comparison. Semin Arthritis Rheum. (2020) 50(3):515–20. 10.1016/j.semarthrit.2020.01.013 32113838

[44] Shahram F Nadji A Akhlaghi M Faezi ST Chams-Davatchi C Shams H Paediatric Behcet’s disease in Iran: report of 204 cases. Clin Exp Rheumatol. (2018) 36(Suppl 115):135–40. 29998839

[45] Nanthapisal S Klein NJ Ambrose N Eleftheriou D Brogan PA. Paediatric Behcet’s disease: a UK tertiary centre experience. Clin Rheumatol. (2016) 35:2509–16. 10.1007/s10067-016-3187-z 26833394 PMC5031738

[46] Atmaca L Boyvat A Yalcindag FN Atmaca-Sonmez P Gurler A. Behcet disease in children. Ocul Immunol Inflamm. (2011) 19(2):103–7. 10.3109/09273948.2011.555592 21428747

[47] Koné-Paut I Yurdakul S Bahabri SA Shafae N Ozen S Ozdogan H Clinical features of Behcet’s disease in children: an international collaborative study of 86 cases. J Pediatr. (1998) 132(4):721–5. 10.1016/S0022-3476(98)70368-3 9580778

