# Supplementary material for: Can early-onset acquired demyelinating syndrome (ADS) hide pediatric Behcet's disease? A case report
Source: Front Pediatr. 2023 Jun 23;11:1175584. doi: 10.3389/fped.2023.1175584 (PMC10327559; doi:10.3389/fped.2023.1175584)
Supplement: Supplementary file 1 [file Image2.pdf]

Figure S2. Flow-chart of the diagnostic approach to pediatric patients with a first neurological episode suggestive for demyelinating disease.

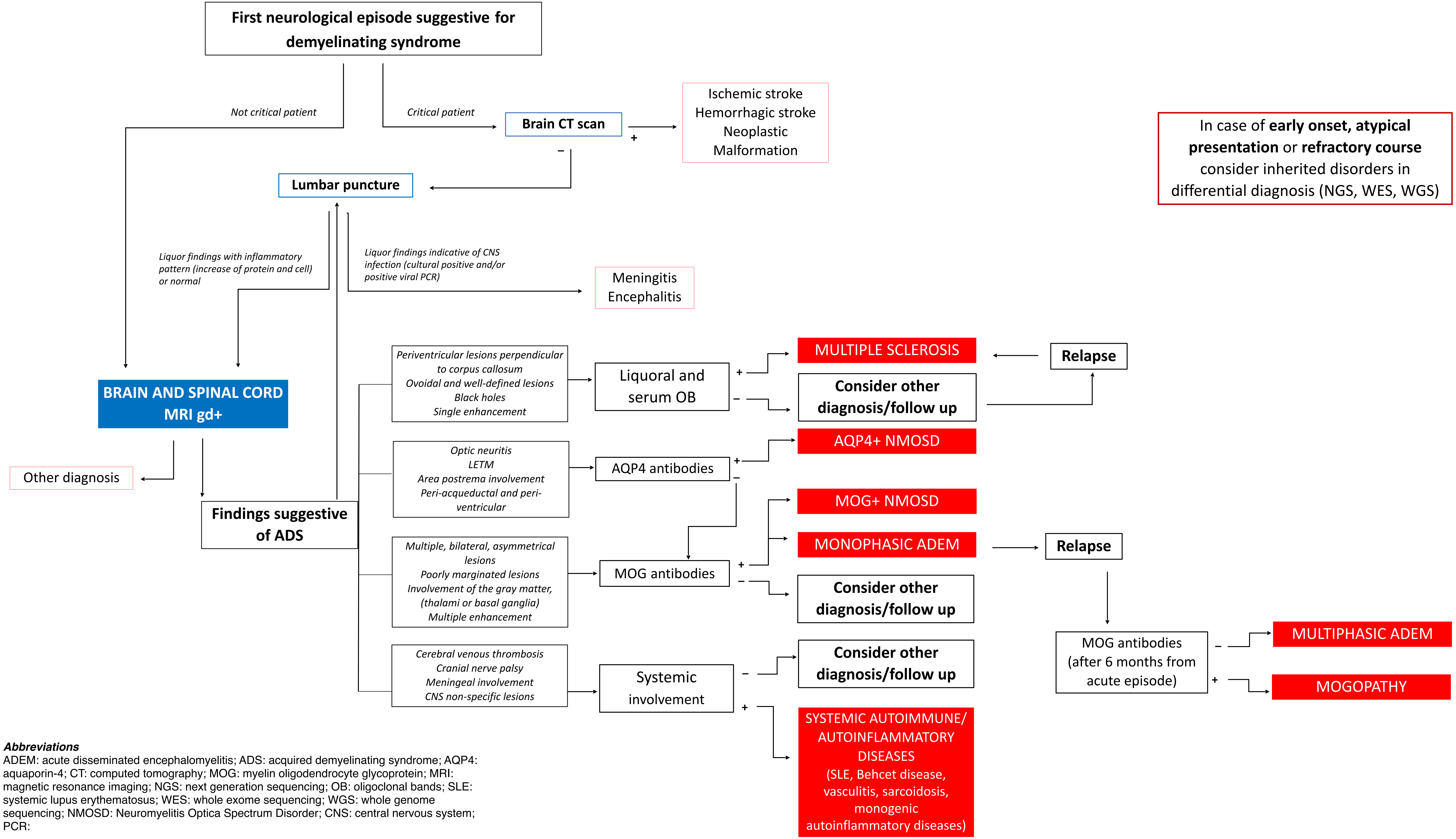

Abbreviations

ADEM: acute disseminated encephalomyelitis; ADS: acquired demyelinating syndrome; AQP4: aquaporin-4; CT: computed tomography; MOG: myelin oligodendrocyte glycoprotein; MRI: magnetic resonance imaging; NGS: next generation sequencing; OB: oligoclonal bands; SLE: systemic lupus erythematosus; WES: whole exome sequencing; WGS: whole genome sequencing; NMOSD: Neuromyelitis Optica Spectrum Disorder; CNS: central nervous system; PCR:
