# Supplementary material for: Can early-onset acquired demyelinating syndrome (ADS) hide pediatric Behcet's disease? A case report
Source: Front Pediatr. 2023 Jun 23;11:1175584. doi: 10.3389/fped.2023.1175584 (PMC10327559; doi:10.3389/fped.2023.1175584)
Supplement: Supplementary file 2 [file Table1.pdf]

Table S1. Concentration of cytokines, chemokines and growth factors measured by multiplex immunoassays in serum and cerebrospinal fluid of the patient at 13 and 19 months. Proteins with increased levels compared to pediatric reference values are highlighted in bold , except for IL-17, FGF and VEGF (pediatric reference values not available).

Observed concentration (pg/ml)	Serum		Cerebrospinal fluid	
Age	**13 months**	**19 months**	**13 months**	**19 months**
IL-1β	6.93	3.73	0.00	0.00
IL-1ra	**2132.87**	**627.19**	**121.40**	**80.23**
IL-2	4.64	5.10	0.00	0.00
IL-4	12.04	9.29	0.26	0.52
IL-5	0.00	10.63	11,44	8,30
IL-6	11.37	4.60	6.40	1.88
IL-7	**64.98**	**64.16**	0.00	0.00
IL-8	**62.10**	**32.86**	7.42	12.50
IL-9	**447.41**	**438.67**	3.46	5.11
IL-10	13.40	3.61	1.86	2.42
IL-12p70	0.81	6.66	0.00	0.00
IL-13	8.35	3.39	2.85	3.76
IL-15	**224.08**	**260.92**	**118.59**	**123.07**
IL-17A	26.12	18.17	0.94	0.85
Eotaxin	**137.50**	**104.98**	0.27	0.35
FGF basic	76.59	63.22	0.00	0.00
G-CSF	**345.14**	**261.85**	**27.55**	**53.36**
GM-CSF	10.22	6.83	0.95	0.85
IFN- γ	9.45	6.47	1.64	1.79
IP-10/ CXCL10	**4474.55**	**503.69**	82.65	165.77
MCP-1/ MCAF	**453.05**	**352.31**	150.42	159.32
MIP-1α	7.64	5.45	0.68	1.70
MIP-1β	**365.86**	**350.61**	4.34	15.25
RANTES	**23640.37**	**29703.75**	5.12	6.01

TNF-α	**146.07**	**117.44**	1.57	3.04
VEGF	237.09	235.01	134.38	121.11

Abbreviations. IL: Interleukin; FGF: Fibroblast Growth Factor; G-CSF: Granulocyte colony stimulating factor; GM-CSF: Granulocyte-macrophage colony–stimulating factor; IFN: Interferon; IP-10: Interferon gamma-induced protein 10/CXCL10: C–X–C motif chemokine 10; MCP-1: monocyte chemoattractant protein-1/MCAF: Monocyte chemotactic and activating factor; MIP: Macrophage Inflammatory Protein; RANTES: Regulated on Activation, Normal T cell Expressed and Secreted; TNF: Tumor Necrosis Factor; VEGF: Vascular-Endothelial Growth Factor

Reference: Pranzatelli MR, Tate ED, McGee NR, Colliver JA. Pediatric reference ranges for proinflammatory and anti-inflammatory cytokines in cerebrospinal fluid and serum by multiplexed immunoassay. J Interferon Cytokine Res. 2013 Sep;33(9):523-8.
