# Supplementary figures and images for: Can early-onset acquired demyelinating syndrome (ADS) hide pediatric Behcet's disease? A case report
Source: Front Pediatr. 2023 Jun 23;11:1175584. doi: 10.3389/fped.2023.1175584 (PMC10327559; doi:10.3389/fped.2023.1175584)

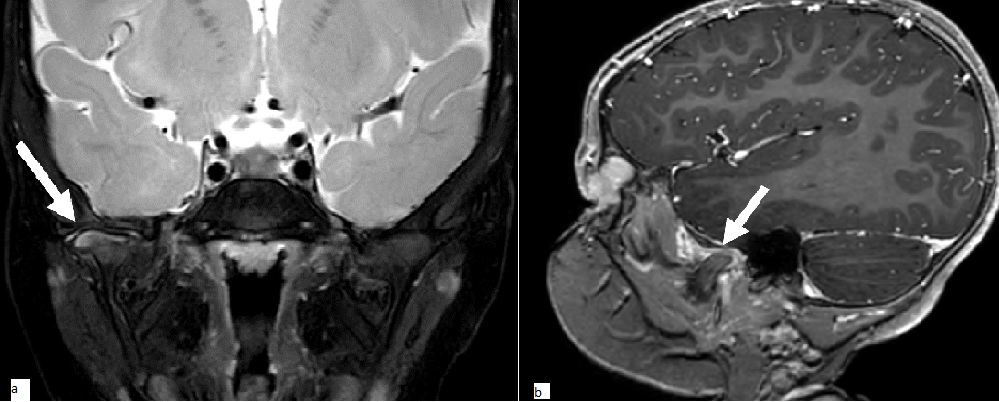

Supplement: Supplementary file 3 [file Image1.jpeg]
